# Prevalence of comorbid autoimmune diseases and antibodies in newly diagnosed multiple sclerosis patients

**DOI:** 10.1186/s42466-024-00351-2

**Published:** 2024-11-12

**Authors:** Konstantin Fritz Jendretzky, Lisa-Marie Lezius, Thea Thiele, Franz Felix Konen, André Huss, Lena Heitmann, Yunus Emre Güzeloglu, Philipp Schwenkenbecher, Kurt-Wolfram Sühs, Jelena Skuljec, Mike Peter Wattjes, Torsten Witte, Christoph Kleinschnitz, Refik Pul, Hayrettin Tumani, Stefan Gingele, Thomas Skripuletz

**Affiliations:** 1 Department of Neurology, Hannover Medical School, Hannover, Germany; 2 Department of Rheumatology and Clinical Immunology, Hannover Medical School, Hannover, Germany; 3 Department of Neurology, University Hospital of Ulm, Ulm, Germany; 4 Department of Neurology and Center for Translational Neuro- and Behavioral Sciences (C-TNBS), University Medicine Essen, Essen, Germany; 5 Department of Neuroradiology, Charité Berlin, Corporate Member of Freie Universität zu Berlin, Humboldt-Universität zu Berlin, erlin, Germany

**Keywords:** Multiple sclerosis, Autoimmune diseases, Prevalence, Comorbidity, Neurofilament light

## Abstract

**Background:**

Diagnosing multiple sclerosis (MS) is challenging due to diverse symptoms and the absence of specific biomarkers. Concurrent autoimmune diseases (AID) or non-specific antibodies further complicate diagnosis, progression monitoring, and management. Data on AID prevalence in MS patients are sparse. This study aims to identify concurrent AIDs alongside MS.

**Methods:**

In this retrospective single-center study, we analyzed patient records at our university hospital from 2010 to 2017, focusing on cases suspected of inflammatory demyelinating disease. The 2017 McDonald criteria were applied. Additionally, we measured neurofilament light (NfL) levels from available CSF samples in our biobank.

**Results:**

We identified a total of 315 patients, of whom 66% were women. In total, 13.7% of all patients had concurrent AID, while 20.3% had isolated antibody findings without AID. The most common AID was autoimmune thyroiditis (8.9%), followed by chronic inflammatory skin diseases (1.6%), arthritis (1%), type 1 diabetes (1%), Sjögren’s syndrome (0.6%), and inflammatory bowel diseases (0.6%). Cardiolipin antibodies were the most frequent isolated antibody finding (8.6%). Our data showed that, from the perspective of the initial demyelinating event, neither comorbid AID nor isolated antibodies significantly influenced relapses or MS progression over a median follow-up of 9 months. Standard CSF parameters and NfL levels were similar between the groups at the time of MS diagnosis.

**Conclusion:**

Our study shows that AIDs, particularly autoimmune thyroiditis, frequently occur at the onset of MS. The proportion of AIDs commonly treated with immunomodulatory therapy in our cohort was similar to that observed in the general population. Comorbid AID did not affect NfL levels, indicating similar disease activity. Future research should explore new AID emergence during the course of MS, especially considering the increased incidence of rheumatic diseases later in life.

**Supplementary Information:**

The online version contains supplementary material available at 10.1186/s42466-024-00351-2.

## Background

Multiple sclerosis (MS) is the most frequent chronic inflammatory demyelinating disease of the central nervous system (CNS) in young adults leading to long term disability [1–3]. The diagnosis is based on the McDonald criteria, initially developed in 2001 and subsequently revised three times [4–7]. The latest revision in 2017 enables earlier diagnosis, potentially even at the first manifestation of the disease, while maintaining high accuracy, as demonstrated in subsequent studies that applied the criteria to various cohorts [7–9]. Despite advancements in diagnostic criteria, the diverse locations of lesions contribute to highly heterogeneous clinical symptoms in MS, presenting challenges for early and accurate diagnosis in some cases [3, 10, 11]. Additionally, the absence of a specific biomarker for MS diagnosis further increases the challenge and carries the risk of misdiagnosis, such as mistaking an underlying rheumatologic disease [11–13].

Conclusive evidence suggests that patients with an existing autoimmune disease (AID) are more prone to developing additional AID, possibly due to a similar pathogenesis [14, 15]. Consequently, MS patients are at higher risk of developing other AIDs affecting various organs and tissues, further complicating diagnosis [15–18]. Therefore, comprehensive consideration of differential diagnoses is essential [7]. As part of this work-up, common antibodies are often tested to clarify possible other diseases [19, 20]. However, the fact that antibody findings do not always correspond to a specific diagnosis means that MS patients often present with isolated antibody findings (ABF) of unclear significance. The relevance of these antibodies needs to be evaluated throughout the disease course and may need to be taken into account when deciding treatment options [21–24]. Concerning the frequency and types of concomitant AID and ABF in MS patients, particularly from before the 2017 revision of the diagnostic criteria, data are often contradictory and partly outdated [14, 18, 24]. Specifically, there is a lack of data on commonly used and newer diagnostic parameters in MS evaluation. Thus, the aim was to identify concurrent AIDs at the onset of MS through record review and standard serological screenings.

## Patients and methods

### Patients’ characteristics

We conducted a retrospective analysis of medical records from patients admitted to the Department of Neurology at Hannover Medical School (MHH; Hannover, Germany) between 2010 and 2017. We focused on patients presenting with symptoms suggestive of a first demyelinating event. The 2017 McDonald criteria were retrospectively applied to the patient cohort, and those diagnosed with MS were included for further analysis. This group consisted in part of patients who had already been described previously [25–27]. Recent criteria were applied to identify patients with neuromyelitis optica spectrum disorder (NMOSD) [28] or with myelin oligodendrocyte glycoprotein antibody-associated disease (MOGAD) [29, 30]. Patients diagnosed with these conditions were excluded from this study.

**Fig. 1:**
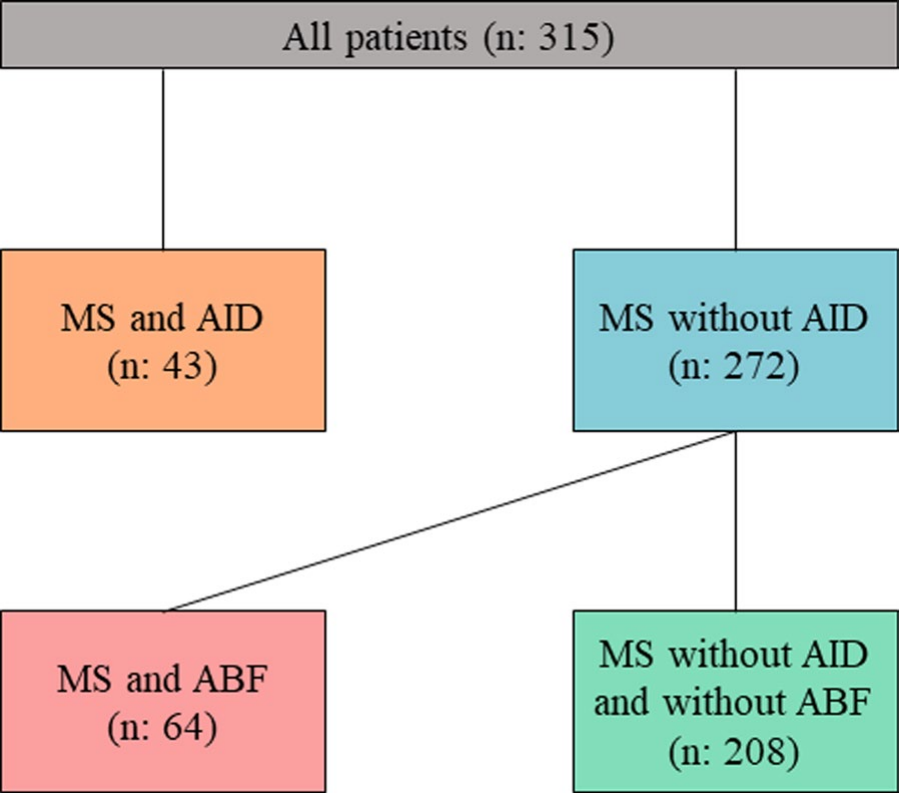
Schematic flowchart of the number of multiple sclerosis (MS) patients and their further categorization into subgroups according to the presence of comorbid autoimmune diseases (AID) or isolated antibody findings (ABF)

Information on concomitant AIDs at the initial manifestation of MS was obtained from the patient files. As part of routine diagnostics, the serological screening included tests for antinuclear antibodies (ANA), anti-neutrophil cytoplasmic antibodies (ANCA), extractable nuclear antigens (ENA), thyroid autoantibodies like TSH receptor antibodies (TRAb) and thyroid peroxidase antibodies (TPOAb), along with cardiolipin, alpha fodrin IgA and IgG, and anti-double stranded DNA antibodies. We divided patients into those with or without a concomitant AID (“MS and AID” and “MS without AID”). Patients without concomitant AID were further divided into those with and without isolated ABF (Fig. 1). Further inclusion criteria were the presence of paired cerebrospinal fluid (CSF) and serum analyses, as well as a brain magnetic resonance imaging (MRI) scan. To categorize the types of initial clinical manifestations, eight distinct groups were identified: 1: optic neuritis, 2: cortical manifestation with paresis, 3: cortical manifestation with exclusively sensory symptoms, 4: infratentorial symptoms, 5: spinal manifestation with paresis, 6: spinal manifestation with exclusively sensory symptoms, 7: polysymptomatic and 8: patients with no current symptomatic.

To analyze the choice and influence of disease-modifying therapies (DMTs), groups were categorized based on the efficacy of the medication: (0) no DMT, (1) low efficacy DMTs (interferon beta, glatiramer acetate, dimethyl fumarate, teriflunomide, azathioprine, methotrexate), (2) intermediate efficacy DMT (fingolimod), and (3) high efficacy DMTs (alemtuzumab, natalizumab, rituximab). This study was approved by the institutional ethics committee (no. 8172-BO-K-2018).

### CSF and serum analytical procedures

CSF and serum samples were routinely analyzed in the Neurochemistry Laboratory of the Department of Neurology at Hannover. The high standards of the analysis’s precision were assured by the regular participation of the laboratory members in the INSTAND external quality assessment program for analytical methods [31]. The cell count was determined microscopically using a Fuchs-Rosenthal counting chamber, and the total protein amount (limit = 500 mg/L) was measured using a Bradford dye-binding method. CSF lactate was determined enzymatically with the cut-off value at 3.5 mmol/l. Immunoglobulin G (IgG), immunoglobulin A (IgA), immunoglobulin M (IgM) and albumin were determined by kinetic nephelometry (Beckman Coulter IMMAGE, Brea, CA, USA), whereas quantitative intrathecal IgG synthesis was calculated according to Reiber-Felgenhauer [32]. The oligoclonal bands (OCB) in the CSF were determined by isoelectric focusing on polyacrylamide gels with subsequent silver staining. Following the first European consensus on CSF analysis in MS, five different OCB patterns were distinguished. At least two apparent bands isolated in CSF were considered as positive result [33]. After routine diagnostics, all samples were stored at -80 degrees.

Neurofilament light chain (NfL) levels in 1:2 diluted CSF samples were determined using the NF-light® ELISA kit (Uman Diagnostics AB, Umea, Sweden), according to the manufacturer’s instructions. The intra- and inter- assay coefficients of variability (CV) were determined by analyzing a pool of CSF five times on each plate. The intra-assay CVs were < 5% and the inter-assay CV was < 10%.

### Statistics

The data was analyzed using GraphPad Prism 8.43 (GraphPad Software, USA) and SPSS (version 26, Armonk, NY, USA). The D’Agostino & Pearson omnibus normality test was used to test for Gaussian distribution. Kruskal–Wallis test and the Dunn post-hoc test were used to compare three or more groups with non-parametric distribution. For the comparison of two groups in non-parametric study samples, the Mann–Whitney U test was performed. One-way analysis of variance with Tukey’s Multiple Comparison Test was used for group comparison in normally distributed samples. Fisher’s exact test was used to measure the independence of two categorical variables. To analyze the influence of age, sex, and group classification on the occurrence of relapses within the determined follow-up period, Cox regressions were conducted. A median split was performed to account for age. To evaluate potential factors influencing Expanded Disability Status Scale (EDSS) worsening during the follow-up period, a binomial regression was conducted. *P* values < 0.05 were considered statistically significant.

## Results

### Demographic and clinical characteristics

In a total cohort of 315 study participants, the median age was 33 years (IQR25-75%: 26—42), and 66% (n = 208) were women. Among all MS patients, 13.7% (n = 43) had a comorbid AID. ABF without a concomitant AID were present in 20.3% (n = 64) of MS patients. Accordingly, we classified patients into the following groups: (i) MS with AID, (ii) MS without AID, (iii) MS with ABF, and (iv) MS without AID and ABF. The clinical severity determined using the EDSS was obtained for all patients.

Eight groups with different clinical manifestations were formed to compare them with the findings of comorbid AID and ABF (Table 1). When comparing the demographic and clinical data a significantly higher proportion of women was found in the group MS and ABF, compared to the group MS without AID and without ABF (76.6% vs. 61.2%; *p* = 0.036). However, when testing each group against the overall cohort, there were no detectable significant differences. Furthermore, a significant difference was found within the group of patients with a spinal manifestation featuring exclusively sensory symptoms, with a higher proportion in the MS with ABF group compared to the MS without AID and without ABF group (MS with ABF: 23.4% vs. MS without AID and without ABF: 10.6%, *p*: 0.0123). All other data did not differ significantly (Table 1).

**Table 1:** Patient characteristics of the cohort

		All	MS and AID	MS without AID	MS without AID but ABF	MS without AID and without ABF
N (%)		315 (100)	43/315 (13.7)	272/315 (86.3)	64/272 (23.5)	208/272 (76.5)
Age (years), median (IQR 25%-IQR 75%)		33 (26–42)	34 (26–44.25)	32.5 (26–42)	33 (26–41)	32 (26–42)
Sex: female, n (%)		208/315 (66)	30/43 (69.8)	178/272 (65.4)	49/64 (76.6)	129/208 (62)
EDSS, median (IQR 25%-IQR 75%)		2 (1.5–2.5)	2.25 (1.625–3)	2 (1.5–2.5)	2 (1–3)	2 (1.5–2.5)
Clinical manifestation form n (%)	1	119 (37.8)	18 (41.9)	101 (37.1)	19 (29.7)	82 (39.4)
	2	8 (2.5)	0 (0)	8 (2.9)	2 (3.1)	6 (2.9)
	3	29 (9.2)	7 (16.3)	22 (8.1)	2 (3.1)	20 (9.6)
	4	52 (16.5)	4 (9.3)	48 (17.7)	11 (17.2)	37 (17.8)
	5	31 (9.8)	3 (7)	28 (10.3)	8 (12.5)	20 (9.6)
	6	43 (13.7)	6 (13.9)	37 (13.6)	15 (23.4)	22 (10.6)
	7	28 (8.9)	5 (11.6)	23 (8.5)	4 (6.3)	19 (9.1)
	8	5 (1.6)	0 (0)	5 (1.8)	3 (4.7)	2 (1)

Clinical manifestation forms were categorized as following: 1: optic neuritis, 2: cortical manifestation with paresis, 3: cortical manifestation with exclusively sensory symptoms, 4: infratentorial symptoms, 5: spinal manifestation with paresis, 6: spinal manifestation with exclusively sensory symptoms, 7: polysymptomatic and 8: patients with no current symptomatic.

*IQR*, interquartile range; MS, multiple sclerosis; *AID*, autoimmune disease; *ABF*, isolated antibody finding; *EDSS*, expanded disability status scale

### Frequency and distribution of AIDs

**Fig. 2:**
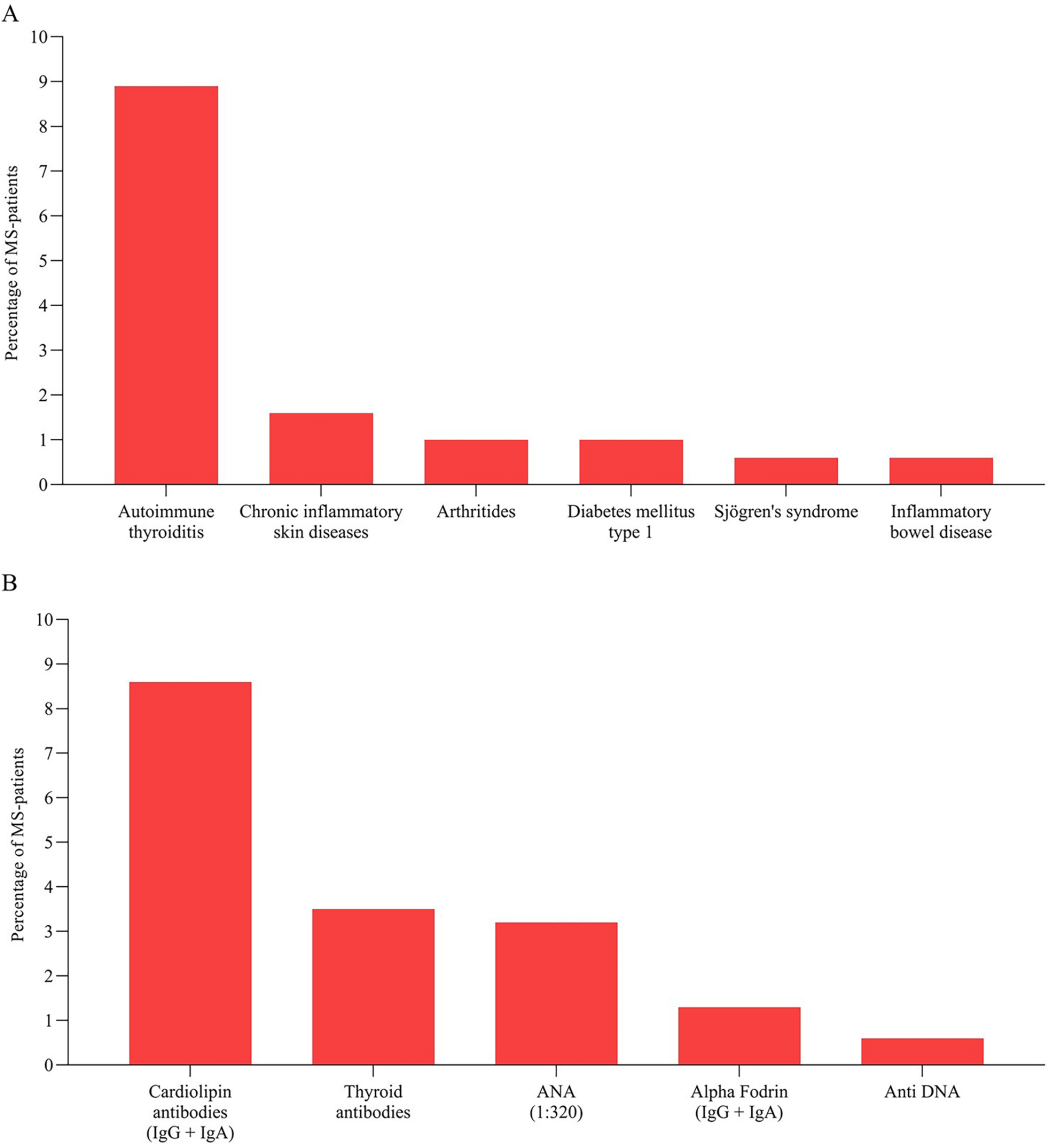
Frequencies of comorbid autoimmune diseases (**A**). Frequencies of antibody findings in the absence of corresponding disease diagnosis. Thyroid autoantibodies were TSH receptor antibodies (TRAb) and thyreoperoxidase antibodies (TPOAb), cardiolipin and alpha fodrin antibodies consisted of immunoglobulin G (IgG) and immunoglobulin A (IgA) antibodies (**B**). ANA, antinuclear antibodies; anti-DNA, anti-double stranded deoxyribonucleic acid antibodies

Among the patients who had a comorbid AID at the time of diagnosis, autoimmune thyroiditis was the most prevalent, affecting 8.9% of MS patients (n = 28). Other observed AIDs included chronic inflammatory skin diseases (n = 5, 1.6%), arthritis (n = 3, 1%), type 1 diabetes (n = 3, 1%), Sjögren’s syndrome (n = 2, 0.6%), and inflammatory bowel diseases (n = 2, 0.6%; Fig. 2A).

### Frequency and distribution of ABF

74% of all MS patients (n = 233) had a detectable ANA titer, generally indicating an isolated ABF. When using a positivity threshold of 1:320, the proportion of positive patients decreased to 3.2% (n = 10).

Using a threshold of 1:320 for ANA titers, ABF without concomitant AID were present in 20.3% (n = 64) of MS patients. Among all tested antibodies, those specific for cardiolipin were the most frequently detected (8.6% of all patients, n = 27). These cardiolipin-specific antibodies were in 22 cases IgM antibodies and in 4 cases IgG antibodies (IgM: 7%, IgG: 1.3% of all MS patients). One patient had both IgM and IgG antibodies (0.3%).

Other ABF included thyroid autoantibodies, found in 3.5% (n = 11) without a diagnosis of autoimmune thyroiditis, alpha fodrin antibodies in 1.3% (n = 4), and anti-DNA antibodies in 0.6% (n = 2). With regard to the alpha fodrin antibodies, the subclasses were divided into 1 patient with alpha fodrin IgA antibodies (0.3%), 2 patients with IgG antibodies (0.6%) and 1 patient with IgA and IgG antibodies (0.3%). In 1 (0.3%) MS patient with no known or newly diagnosed autoimmune disease, a positive ENA screening was obtained, with the exact antibody finding being an anti-U1RNP antibody finding. In 9 patients (2.9%) more than one specific ABF, each without correlating AID was present. (Fig. 2B).

### Influence of AID and ABF on laboratory parameters

Next, we evaluated the potential impact of comorbid AID and ABF on diagnostic parameters in serum and CSF of the included patients.

Regarding the standard CSF parameters, such as cell count, lactate, the frequency of oligoclonal bands, and the presence of intrathecal IgG, IgA, and IgM synthesis, there were no significant differences between the groups (Table 2).

**Table 2:** Comparison of commonly used laboratory parameters between the described groups

Parameters	MS and AID	MS without AID	MS and ABF	MS without AID and ABF
Cells > 4, % (n)	55.8 (24/43)	67.6 (184/272)	65.6 (42/64)	68.3 (142/208)
Cells per µl, median (IQR 25%—IQR 75%)	5.2 (2.8–11)	7 (3–13.9)	6.2 (3–13)	7 (3–14)
CSF lactate (mmol/l), median (IQR 25%—IQR 75%)	1.6 (1.5–2)	1.8 (1.6–2.2)	1.7 (1.5–2.1)	1.8 (1.6–2.2)
OCB positivity, % (n)	100 (43/43)	98.2 (267/272)	98.4 (63/64)	98.6 (205/208)
Presence of intrathecal IgG synthesis, % (n)	67.4 (29/43)	61.4 (167/272)	64.1 (41/64)	60.1 (125/208)
Presence of intrathecal IgA synthesis, % (n)	11.6 (5/43)	10.3 (28/272)	10.9 (7/64)	10.1 (21/208)
Presence of intrathecal IgM synthesis, % (n)	37.2 (16/43)	29.4 (80/272)	29.7 (19/64)	29.3 (61/208)

IQR, interquartile range; OCB, oligoclonal bands; IgG, immunoglobulin G; IgA, immunoglobulin A; IgM, immunoglobulin M; MS and AID, multiple sclerosis patients with comorbid autoimmune disease; MS without AID, multiple sclerosis patients without comorbid autoimmune disease; MS and ABF, multiple sclerosis patients with isolated antibody finding; MS without ABF and without AID, multiple sclerosis patients without isolated antibody finding or comorbid autoimmune disease

**Fig. 3:**
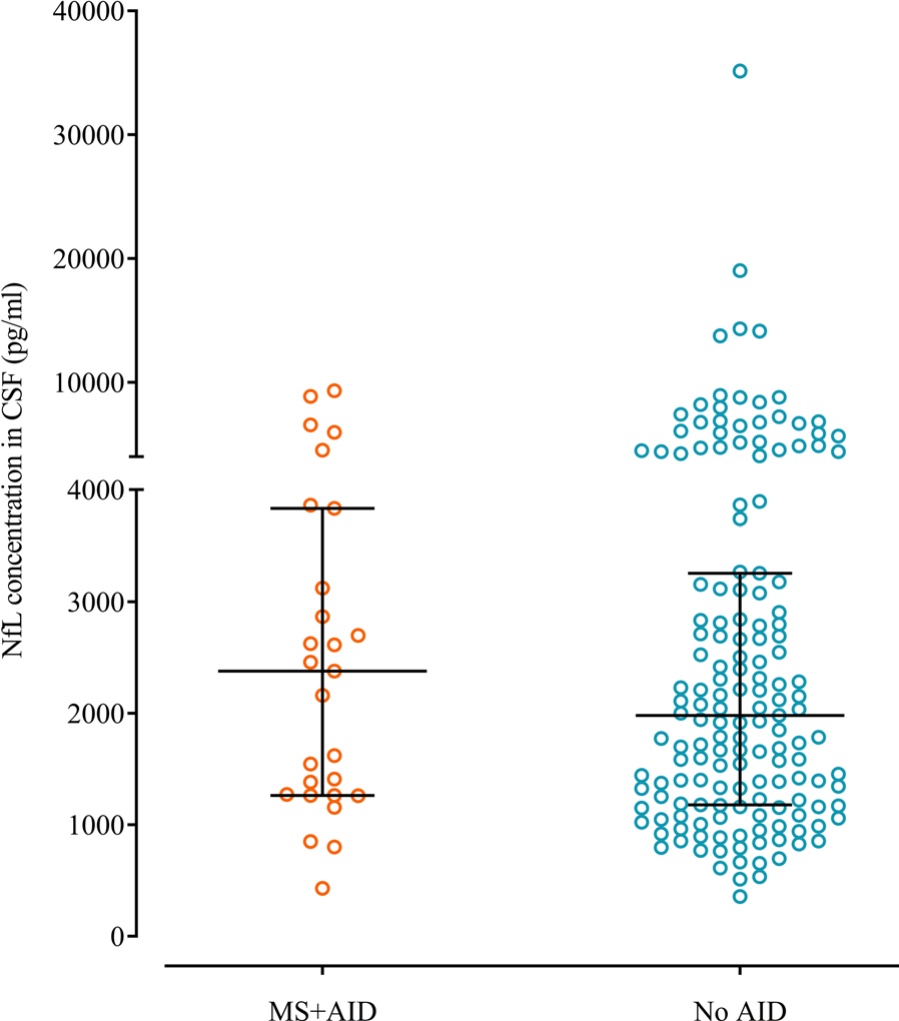
Comparison of the measured CSF NFL concentration between the groups. Lines and error bars indicate median ± interquartile range. NFL, neurofilament light; CSF, cerebrospinal fluid; MS and AID, multiple sclerosis patients with comorbid autoimmune disease; MS without AID, multiple sclerosis patients without comorbid autoimmune disease

Similarly to standard diagnostic parameters, also levels of intrathecal NFL did not differ between the groups (median MS and AID: 2378 pg/ml, MS without AID: 1979 pg/ml, MS and ABF: 1697 pg/ml, MS without AID and ABF: 2060 pg/ml; Fig. 3).

### Influence of AID and ABF on the MS disease course

To investigate whether AID or ABF can increase the number of clinical events before MS diagnosis, we calculated the number of relapses detected in each patient group. Disease activity indicated by clinical events before MS was diagnosed did not significantly differ between the groups (MS with AID, MS without AID, MS with ABF, MS without AID and ABF) (Supplemental Table 1).

Follow-up data was available for 66.7% (210 out of 315) of all MS patients, with a median follow-up duration of 9 months (IQR 25%: 2.5 months, IQR 75%: 27 months). Among these patients, 28 (13.3%) were classified in the MS + AID group, while 182 (86.7%) fell into the MS without AID group. Additionally, 45 patients (21.4%) were categorized as MS + ABF, and 137 patients (65.2%) as MS without AID and without ABF. To analyze the selection and impact of DMTs, groups were formed based on low, intermediate, and high efficacy categories, as described in the methods section. Sufficient data was available for 157 patients (74.8% of those with follow-up). The analysis revealed no significant differences in the choice of DMTs between the groups (Table 3).

**Table 3:** Choice of therapy in followed up patients

Type of disease modifying therapy	All (n:157)	MS and AID (n:24)	MS without AID (n:133)	MS and ABF (n: 35)	MS without AID and without ABF (n: 98)
None, n (%)	30 (19.1)	3 (12.5)	27 (20.3)	9 (25.7)	18 (18.4)
Low efficacy, n (%)	115 (73.2)	18 (75)	97 (72.9)	24 (68.6)	73 (74.5)
Intermediate efficacy, n (%)	5 (3.2)	2 (8.3)	3 (2.3)	1 (2.9)	2 (2)
High efficacy, n (%)	7 (4.5)	1 (4.2)	6 (4.5)	1 (2.9)	5 (5.1)

AID, autoimmune disease; ABF, isolated antibody finding; low potency therapy: azathioprine, methotrexate, teriflunomide, dimethyl fumarate, interferon beta and glatiramer acetate; intermediate potency: fingolimod; high potency disease-modifying therapies: alemtuzumab, rituximab, natalizumab

Upon closer examination of the comorbid AID that could be relevant as secondary therapeutic targets, no clear pattern of therapeutic choice was observed. Among the five patients with a comorbid chronic skin condition, follow-up data were available for two, both of whom were treated with dimethyl fumarate. Among the three patients with arthritis, two received interferon beta, and one was treated with methotrexate. Of the two patients with inflammatory bowel disease, one received glatiramer acetate, and the other was treated with interferon beta. For the two patients with comorbid Sjögren’s syndrome, one was treated with teriflunomide, while the other received interferon beta.

Cox regression analyses were conducted to assess whether relapses were more likely to occur in one of the defined groups, taking into account age, sex, and treatment efficacy. In both analyses, age was a significant predictor (MS + AID vs. MS without AID: *p* < 0.001; HR: 0.955, 95% CI 0.930–0.980; MS + ABF vs. MS without AID and without ABF: *p* = 0.006; HR: 0.956, 95% CI 0.926–0.987). Additionally, the first analysis indicated that the presence of AID significantly increased the risk of a relapse event (*p* = 0.023; HR = 1.938, 95% CI 1.097–3.425), whereas the presence of ABF significantly reduced the risk of a relapse event during follow-up (p = 0.023; HR: 0.428, 95% CI 0.206–0.890).

To further analyze the data with adjustment for age, we performed separate analyses stratified by median age (≤ 32 years and > 32 years). This revealed that the presence of ABF again acted as a risk-reducing factor compared to the MS without AID and ABF group (*p* = 0.013; HR = 0.295, 95% CI 0.112–0.775), although this was only evident within the younger group. In the same subgroup, treatment with intermediate efficacy DMTs was associated with an increased risk of a relapse event, although a very wide confidence interval was observed (*p* = 0.013; HR = 22.575, 95% CI 1.938–263.015). All other factors did not differ significantly in both analyses, and the previously observed elevated risk in the AID vs. MS without AID group was no longer detected in the age-adjusted analyses (see supplemental Table 2).

Moreover, both EDSS at the time of the last follow-up (median EDSS MS and AID: 1.5, MS without AID: 1.25, MS with ABF: 1, MS without AID and ABF: 1.5) and differential EDSS, calculated as the difference between the last and first EDSS recorded (median EDSS MS and AID: − 0.25, MS without AID: 0, MS and ABF: 0, MS without AID and ABF: -0.25), did not demonstrate any significant differences in the MS patients having comorbid AID or ABF, compared to those without (Supplemental Fig. 1A and B, respectively).

In an evaluation of newly identified autoimmune diseases during follow-up, 5 out of the 210 patients (2.4%) were affected. In the first case, a patient with a previously known diagnosis of Hashimoto’s thyroiditis was additionally diagnosed with antiphospholipid antibody syndrome during follow-up. In the second case, Sjögren’s syndrome was diagnosed in a patient who also had a pre-existing diagnosis of Hashimoto’s thyroiditis. In the remaining three cases, no autoimmune disease was previously known. Among these, one patient was diagnosed with Crohn’s disease, another with Hashimoto’s thyroiditis, and the last patient was diagnosed with parietal cell antibody-positive autoimmune gastritis as well as type 1 diabetes mellitus.

## Discussion

The diagnosis of MS can be challenging in neurological routine mainly due to its heterogeneous manifestations and the lack of a specific biomarker [3, 12]. It has been suggested that concomitant AIDs and ABFs of unclear significance may impact the diagnosis and the disease course of MS [16, 34]. We were able to show here that with 13% of all MS patients the prevalence of an AID was considerably high. Furthermore, regarding the prevalence of isolated ABF, a higher frequency was observed in our MS cohort compared to other studies on healthy individuals, with notably elevated levels of ANA, irrespective of the diagnostic threshold applied [36]. However, among the patients who had a comorbid AID at the time of diagnosis, autoimmune thyroiditis was the most prevalent, affecting 8.9% of MS patients. Along with type 1 diabetes (1%), both of these AID are typically not treated with immunomodulatory therapies. When focusing solely on AID that are treated with immunomodulatory therapies -such as inflammatory skin diseases, arthritis, Sjögren’s syndrome, and inflammatory bowel diseases- the proportion was 5%. This is comparable to the 4% prevalence observed in the general population, as reported by the Central Institute for National Health Care in the Federal Republic of Germany during the same period [35].

Our analyses showed that the presence of AID and isolated ABF did not have a significant impact on laboratory parameters in the CSF of MS patients at the time of diagnosis. There were no substantial changes in pleocytosis, intrathecal synthesis of immunoglobulins IgG, IgA, and IgM, or the presence of oligoclonal bands. Moreover, NFL, a newly introduced parameter serving as a marker for neuroaxonal damage and correlating with disease activity, was not found to be increased in the CSF of MS patients at the time of diagnosis, regardless of comorbid AID or isolated ABF. However, it should be noted that NFL levels were measured at the time of the initial demyelinating event, where generally higher values are expected. For more accurate classification, follow-up blood measurements would ideally be necessary, but these were not available in this study.

In addition, consistent with other studies [18, 19, 21], our data suggest that neither AID nor isolated ABF significantly influence the course of MS, as we found no clear differences between the groups regarding MS relapse or disease progression measured by the EDSS. There was some evidence that an ABF might reduce the risk of a relapse event, however, this finding should be interpreted cautiously due to the limited data available and the relatively short follow-up period of 9 months.

The routine testing for antibodies in the diagnostic process for MS has been viewed critically for some time. Some authors and professional societies recommend it only when there is a justified suspicion of an alternative diagnosis [20, 37]. Nevertheless, it remains common practice to conduct parallel diagnostics for possible comorbid AID. However, our data indicate that ABF do not appear to have clinical relevance in most cases during follow up, with only a few new diagnoses of AIDs arising. In addition, experience shows that such findings can lead to uncertainty among patients. Therefore, the practice of broad antibody testing should be critically reconsidered.

The general prevalence of comorbid AID was relatively high in our cohort. However, as previously mentioned, it is important to note that the majority of these AIDs were cases of autoimmune thyroiditis, which are typically treated without immunosuppression. This minimizes the potential interference with MS treatment decisions. In an evaluation of the chosen DMTs, no significant abnormalities were observed, indicating that a tendency toward potentially intensified therapy due to concomitant AID was not evident in our data. Notably, dimethyl fumarate was selected for two patients with psoriasis, primarily because it was also prescribed for treating psoriasis during the inclusion period. However, it is important to note that the data is limited by the fact that the patients were enrolled between 2010 and 2017. We speculate that the increasing emphasis on high-efficacy therapies in the years following enrollment, coupled with the introduction of CD20-depleting therapies and substances such as cladribine to the market, suggests that the results might have been different if recruitment had been performed in later years.

It should be emphasized that the relatively young age of our study cohort might have led to an underrepresentation of specific AID at first manifestation of MS. Especially AID such as rheumatic arthritis typically manifest at a later age and are known to influence therapy choices in MS [16, 34]. For instance, medications commonly used by rheumatologists, such as TNF alpha blockers, may worsen MS [34]. Therefore, because some medications for MS might also be effective for other AID; conversely, they could exacerbate other autoimmune conditions [34], it is crucial not only to investigate the presence of other autoimmune diseases at the onset of MS but also throughout its course. Furthermore, AID could potentially exacerbate symptoms such as fatigue, significantly influencing the course of the disease and patient well-being, making the detection of AID crucial in clinical practice.

## Conclusions

This large, single-center study highlights the significant co-occurrence of AIDs, with autoimmune thyroiditis being the most prevalent during the early stages of MS. Focusing on AIDs that are typically treated with immunomodulatory therapy, the proportion in our cohort was similar to that observed in the general population. Future studies should investigate the occurrence of additional AID during the later course of MS, particularly given the higher prevalence of rheumatic diseases with advancing age.

## Supplementary Information

Additional file 1.

Additional file 2.

Additional file 3.

Additional file 4.

## Data Availability

The datasets used and analyzed during the current study are available from the corresponding author on reasonable request.

## Abbreviations

MS: Multiple sclerosis
NMOSD: Neuromyelitis optica spectrum disorder
MOGAD: Myelin oligodendrocyte glycoprotein antibody-associated disease
AID: Autoimmune disease
ABF: Antibody finding
CSF: Cerebrospinal fluid
IgG: Immunoglobulin G
IgA: Immunoglobulin A
IgM: Immunoglobulin M
NFL: Neurofilament light
CV: Coefficients of variability
OCB: Oligoclonal bands
MRI: Magnetic resonance imaging
TRAb: TSH receptor antibodies
TPOAb: Thyreoperoxidase antibodies
ANA: Antinuclear antibodies
Anti-DNA: Anti-double stranded deoxyribonucleic acid antibodies
ENA: Extractable nuclear antigens
ANCA: Anti-neutrophil cytoplasmic antibodies
EDSS: Expanded disability status scale
