# Supplementary material for: Prevalence of comorbid autoimmune diseases and antibodies in newly diagnosed multiple sclerosis patients
Source: Neurol Res Pract. 2024 Nov 12;6:55. doi: 10.1186/s42466-024-00351-2 (PMC11556020; doi:10.1186/s42466-024-00351-2)
Supplement: Supplementary file 1 — Additional file 1. [file 42466_2024_351_MOESM1_ESM.docx]

**Supplemental Fig 1A:** Comparison of EDSS at last follow up between the groups.

**Supplemental Fig 1B:** Comparison of EDSS difference during follow up between the groups. EDSS difference was calculated by subtracting the first EDSS from the last EDSS recorded.
Lines and error bars indicate median ± interquartile range.
EDSS, Expanded Disability Status Scale; MS and AID, multiple sclerosis patients with comorbid autoimmune disease; MS without AID, multiple sclerosis patients without comorbid autoimmune disease; MS and ABF, multiple sclerosis patients with isolated antibody finding; MS without ABF and without AID, multiple sclerosis patients without isolated antibody finding or comorbid autoimmune disease.
