# Supplementary figures and images for: Prevalence of comorbid autoimmune diseases and antibodies in newly diagnosed multiple sclerosis patients
Source: Neurol Res Pract. 2024 Nov 12;6:55. doi: 10.1186/s42466-024-00351-2 (PMC11556020; doi:10.1186/s42466-024-00351-2)

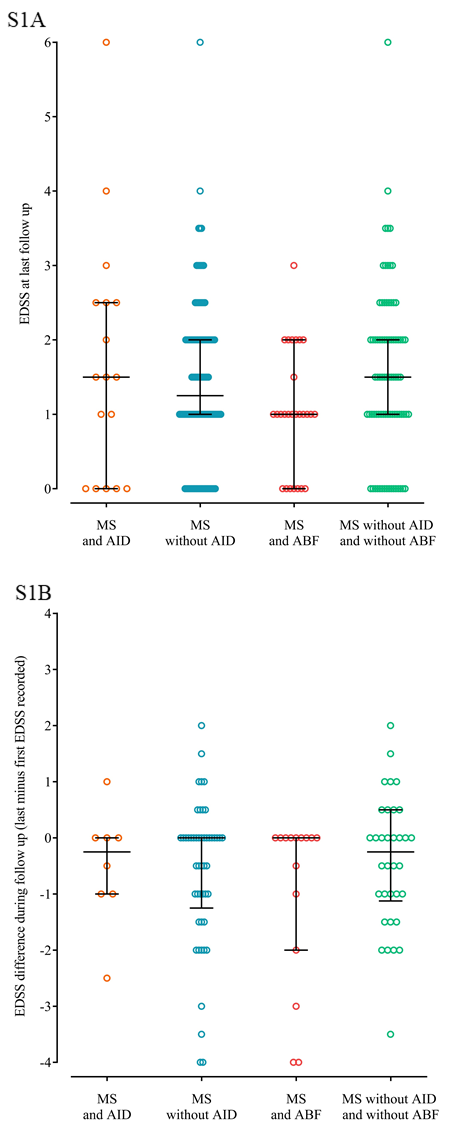

Supplement: Supplementary file 2 — Additional file 2. [file 42466_2024_351_MOESM2_ESM.tif]
