# Supplementary material for: Prevalence of comorbid autoimmune diseases and antibodies in newly diagnosed multiple sclerosis patients
Source: Neurol Res Pract. 2024 Nov 12;6:55. doi: 10.1186/s42466-024-00351-2 (PMC11556020; doi:10.1186/s42466-024-00351-2)
Supplement: Supplementary file 3 — Additional file 3. [file 42466_2024_351_MOESM3_ESM.docx]

| Supplemental table 1: Clinical events before MS diagnosis | | | | |
| --- | --- | --- | --- | --- |
|  | **MS and AID** | **MS without AID** | **MS and ABF** | **MS without AID and ABF** |
| No clinical event before diagnosis of MS, % (n) | 55.8 (24/43) | 69.1 (188/272) | 67.2 (43/64) | 69.7 (145/208) |
| ≥ 1 clinical event before diagnosis of MS, % (n) | 44.2 (19/43) | 30.9 (84/272) | 32.8 (21/64) | 30.3 (63/208) |

**Supplemental table 1:** Occurrence of clinical events before MS diagnosis in patients with or without AID or ABF. MS and AID, multiple sclerosis patients with comorbid autoimmune disease; MS without AID, multiple sclerosis patients without comorbid autoimmune disease; MS and ABF, multiple sclerosis patients with isolated antibody finding; MS without AID and ABF, multiple sclerosis patients without comorbid autoimmune disease and isolated antibody finding.
