# Supplementary material for: Prevalence of comorbid autoimmune diseases and antibodies in newly diagnosed multiple sclerosis patients
Source: Neurol Res Pract. 2024 Nov 12;6:55. doi: 10.1186/s42466-024-00351-2 (PMC11556020; doi:10.1186/s42466-024-00351-2)
Supplement: Supplementary file 4 — Additional file 4. [file 42466_2024_351_MOESM4_ESM.docx]

| Supplemental table 2: Results of respective cox regression analyses regarding clinical outcome in patients followed up | | | | |
| --- | --- | --- | --- | --- |
| Variable, p; hazard ratio (95% CI: Lower - upper) | | **Whole Group** | **Adjusted for age: ≤ 32 years** | **Adjusted for age: > 32 years** |
| MS and AID vs. MS without AID | | | | |
| Age | | **<.001; 0.955 (0.930 - 0.980)** | .052; 0.928 (0.861 - 1.001) | .089; 0.952 (0.899 - 1.008) |
| Sex | | .231; 0.744 (0.459 - 1.207) | .138; 0.631 (0.343 - 1.160) | .867; 1.070 (0.483 - 2.370) |
| Therapy Group (Overall) | | .754 | .340 | .797 |
|  | Weak potency | .966; 0.986 (0.503 - 1.931) | .679; 1.224 (0.470 - 3.183) | .751; 0.849 (0.310 - 2.330) |
|  | Intermediate potency | .694; 1.302 (0.350 - 4.850) | .097; 3.855 (0.782 - 19.009) | .985; 0.000 (N/A - N/A) |
|  | Strong potency | .393; 1.665 (0.517 - 5.365) | .575; 1.636 (0.293 - 9.121) | .491; 1.803 (0.337 - 9.649) |
| Group AID vs. no AID | | **.023; 1.938 (1.097 - 3.425)** | .377; 1.425 (0.650 - 3.125) | .078; 2.367 (0.908 - 6.170) |
| MS and ABF vs. MS without AID and without ABF | | | | |
| Age | | **.006; 0.956 (0.926 - 0.987)** | .292; 0.957 (0.882 - 1.038) | .165; 0.951 (0.886 - 1.021) |
| Sex | | .520; 0.837 (0.488 - 1.437) | .050; 0.486 (0.237 - 0.999) | .478; 1.414 (0.543 - 3.684) |
| Therapy Group | | .962 | .098 | .976 |
|  | Weak potency | .828; 1.082 (0.531 - 2.204) | .502; 1.398 (0.526 - 3.713) | .974; 0.981 (0.313 - 3.080) |
|  | Intermediate potency | .868; 0.833 (0.097 - 7.158) | **.013; 22.575 (1.938 - 263.015)** | .986; 0.000 (N/A - N/A) |
|  | Strong potency | .620; 1.402 (0.369 - 5.332) | .578; 1.643 (0.286 - 9.454) | .686; 1.598 (0.165 - 15.470) |
| Group MS and ABF vs. MS without AID and without ABF | | **.023; 0.428 (0.206 - 0.890)** | **.013; 0.295 (0.112 - 0.775)** | .162; 0.386 (0.101 - 1.468) |

**Supplemental table 2:** Age, sex, therapy group as well as the presence of AID or ABF were included in the analysis. A median split was used for age stratification, resulting in three groups (whole group, ≤ 32 and > 32). Potentially due to limited data, reliable confidence intervals could not be calculated for the subgroup of moderately effective therapies (> 32 years). 95% CI, 95% confidence interval, MS and AID, multiple sclerosis patients with comorbid autoimmune disease; MS without AID, multiple sclerosis patients without comorbid autoimmune disease; MS and ABF, multiple sclerosis patients with isolated antibody finding; MS without AID and ABF, multiple sclerosis patients without comorbid autoimmune disease and isolated antibody finding; N/A, not applicable.
